# Prevalence of JC and BK viruses in Patients with Colorectal Cancer: A Systematic Review and Meta- Analysis

**DOI:** 10.31557/APJCP.2020.21.6.1499

**Published:** 2020-06

**Authors:** Hamid Reza Shoraka, Omid Aboubakri, Ahmad Naghibzadeh-Tahami, Hamid Reza Mollaei, Zohreh Bagherinezhad, Reza Malekpour Afshar, Armita Shahesmaeili

**Affiliations:** 1 *Vector-Borne Diseases Research Center, North Khorasan University of Medical Sciences, Bojnurd, Iran. *; 2 *Faculty of Health, Kerman University of Medical Sciences, Kerman, Iran. *; 3 *Physiology Research Center, Institute of Basic and Clinical Physiology Sciences, Kerman University of Medical Sciences, Kerman, Iran. *; 4 *Research Center of Tropical and Infectious Diseases, Kerman University of Medical Sciences, Kerman. Iran. *; 5 *Librarian, Health faculty, Mazandaran University of Medical Sciences, Mazandaran, Iran. *; 6 *Department of Medical Library and Information Science, Kerman University of Medical Sciences, Kerman, Iran. *; 7 *Neuroscience Research Center, Institue of Neuropharmacology, Kerman University of Medical Sciences, Kerman, Iran. *; 8 *HIV/STI Surveillance Research Center, and WHO Collaborating center for HIV surveillance, Institute for Futures Studies in Health, Kerman University of Medical Sciences, Kerman, Iran. *

**Keywords:** Colorectal neoplasms, JC virus, BK virus, systematic review, meta-analysis

## Abstract

**Introduction:**

Polyomaviruses including BK virus (BKV) and JC virus (JCV) are widespread in human and have been associated with colorectal cancer (CRC) in some studies. The aim of present systematic review and meta-analysis article is to calculate the pooled prevalence of BKV and JCV in patients with CRC and assessing their association with this malignancy.

**Materials and Methods:**

Domestic databases and Sciences Direct, PubMed, ProQuest, Web of Sciences and Scopus were searched for relevant articles up to 2nd June 2019Two independent reviewers extracted the related data from eligible articles. The pooled prevalence and pooled odds ratio (POR) and their 95% confidence interval (95% CI) were calculated using “metaprop” and “metan” commands in Stata 14. Where I2 statistics were >50%, the random effect model was used.

**Results:**

From 1461 relevant studies, 24 articles were eligible and included in the qualitative while 19 articles included in quantitative analysis. The pooled prevalence based on diagnostic methods varies from 29% using immunohistochemistry to 52% using nested-PCR method. The likelihood of being infected with JCV was significantly higher in CRC patients compared to healthy (POR: 4.41, 95% CI: 2.13 – 9.13) controls, normal adjacent mucosa (POR: 2.79, 95% CI: 1.3-5.9) and colorectal adenoma (POR: 3.1, 95% CI: 1.5-6.5) but was not significant when non-CRC patients used as control group.

**Conclusion:**

The prevalence of JCV in colorectal patients was substantially variable by different methods and targets. The significant association between JCV and CRC that was observed in the present study is not indicative of causation and should be studied more in large-scale prospective designs.

## Introduction

Colorectal cancer (CRC) is the third most commonly diagnosed cancer and the second-leading causes of cancer-related mortality in the world (Bray et al., 2018). According to the WHO estimates, it is expected to see more than 60% increase in the incidence and mortality rate of this cancer up to 2030 (Ferlay et al., 2013). Environmental and genetic factors can increase the chance of developing CRC. Although familial aggregation of CRC has been proved well, majority of CRC are sporadic rather than being familial (Lynch and De la Chapelle, 2003). Variety of risk factors including gender, race, obesity, consumption of red and processed meat, tobacco smoking and alcohol consumption has been known to be associated with increased risk of CRC (Liang et al., 2009; Chan et al., 2011). Recently, the relationship between some infectious agents with cancer has been noticed. About one-fifth of cancers are related to infectious factors, mostly viral agents (Höcker and Hohenberger, 2003). For example infection with Epstein- Barr virus has been associated with gastric and nasopharyngeal cancers as well as Burkitt’s lymphoma (Murphy et al., 2009; Shannon-Lowe et al., 2017; Tsao et al., 2017) while human T-lymphotropic virus has been associated with Adult T cell Leukemia. (Bangham and Ratner, 2015).

The role of viral agent in CRC haven’t been studied well. Recently, the association of polyomaviruses with CRC attracted a lot of attention, but the results of studies are inconclusive. The family of polyomaviruses consists of 10 members including BK virus (BKV) and JC virus (JCV). While JCV is observed in 40% of normal colon mucosa, a higher prevalence of JCV (90%) has been observed in colorectal cancer cases (Coelho et al., 2013). Similarly, a relationship has been observed between infection with BKV and CRC (Narayanan et al., 2007; Abend et al., 2009). But different detection methods, study population and choose of control group in these studies resulted in non-identical findings and limits comparability of their results (Theodoropoulos et al., 2005; Sinagra et al., 2014).

The aim of present study is to provide a comprehensive systematic review and meta-analysis of available researches on prevalence of JCV and BK in CRC and provide evidence-based analysis of literature relating these viruses and CRC. As a second object, we also assessd the association between the prevalence of the viruses and colorectal cancer

## Materials and Methods

This systematic review was conducted based on the guidelines provided for Meta-analysis Of Observational Studies in Epidemiology (MOOSE). PRISMA 2009 flow diagram used for detailing the identification and selection of studies for inclusion in the review.

*Search strategy*

We performed automated searches on electronic databases including Sciences Direct, PubMed, ProQuest, Web of Sciences and Scopus up to 2nd June 2019 without restrictions of geographical area and publication date. A search strategy was carefully defined to capture all potentially eligible studies. We used a combination of Medical Subject Headings (MeSH) and similar text words just in English language. Manual search methods were done to identify additional relevant articles. The key MeSH terms combined with the Boolean operator including (“Colorectal Neoplasms OR “Colorectal Tumors OR “Colorectal Carcinoma OR “Colorectal Cancer” OR “Colonic Neoplasms” OR “Rectal Neoplasms” OR “Rectal Tumors” OR “Rectal Cancer” OR “Rectum Cancer”) and (“JC Virus” OR “JC polyomavirus” OR “Polyomavirus hominis 2” OR “John Cunningham Virus” OR “Human Polyomavirus JC” OR “BK Virus” OR “Polyomavirus, BK” OR “Polyomavirus hominis 1” OR “BK polyomavirus” OR “Human Polyomavirus BK”).

*Study selection*

To calculate the prevalence of viruses in CRC, English original studies with interventional and observational designs including cross-sectional, case-control, cohort reported JCV or BKV prevalence (point prevalence) in colorectal cancer patients were included in the review. To measure the strength of association between JCV/BKV and CRC, English original studies reported any of the three measures of association, including odds ratio (OR), relative risk (RR) or prevalence rate ratio (PRR) for JCV and BKV based on tissue of CRC patient, and control group were included. Studies were also included if the OR could be calculated from the data. Studies were excluded if they were: i) review or case report; ii) studies on cell lines; iii) conducted in a selective population like HIV infected patients; iv) not included relevant extractable data; v)used plasma or urine sample. Where more than one publication related to the same study, only the more informative one was used.

*Article screening*

Two independent reviewers (A.N and O.A) carefully scanned all titles, abstracts, and keywords of every published article for their relevancy and eligibility criteria. Any disagreements between the two reviewers resolved by further investigation and discussion among the authors. If the information in the title or abstract was insufficient, the full text was reviewed.Eventually 19 articles were included in meta-analysis.

*Data extraction and management*

The data extraction procedure was performed by two reviewers (H.SH. and A.N.) using a digital data extraction form. Extracted information were: the name of the first author, the study design, the year of publication and characteristics of study population(country and study setting, gender, age and sample size), types of control group(adjacent tissue in the same patient, healthy population, adenoma controls,etc.), virus type(JCV or BKV), types of biologic sample(tissue or plasma), detection methods(PCR, nested PCR, etc.), virus targets and study type. For assurance, the investigators reviewed randomly selected 50% of each other’s reviewed studies and any disagreements between the two reviewers were solved by consulting with a third reviewer (O.A) and pathologist (R.M).

All information was double entered in the data extraction form in order to avoid data entry errors. 

*Assessment of methodological quality*

The quality of articles was evaluated with The Joanna Briggs Institute critical appraisal checklist for prevalence, Cohort and Case Control Studies. Studies assessed according to the following methodological criteria: used valid methods, appropriate sample and appropriate statistical analysis.

*Data analysis*

The pooled prevalence was calculated using the “metaprop” command and the pooled odds ratio (POR) was calculated by “metan” command in Stata software Version 14. The random effects model was employed to estimate the pooled measures and 95% Confidence intervals (95% CI). A two-sided P-value <0.05 of POR was considered statistically significant. The subgroups analysis was performed just in groups with at least three studies. The forest plot was used to display the results of the meta-analysis. The Begg’s funnel plot and egger’ test were draw to evaluate the publication bias of analytical studies.

Furthermore, a meta-regression analysis was applied to investigate the impact of some variables such as type of diagnosis test, location of sampling and sample type on the I^2^, and pooled OR.

## Results

*Literature search result*

**Figure 1:**
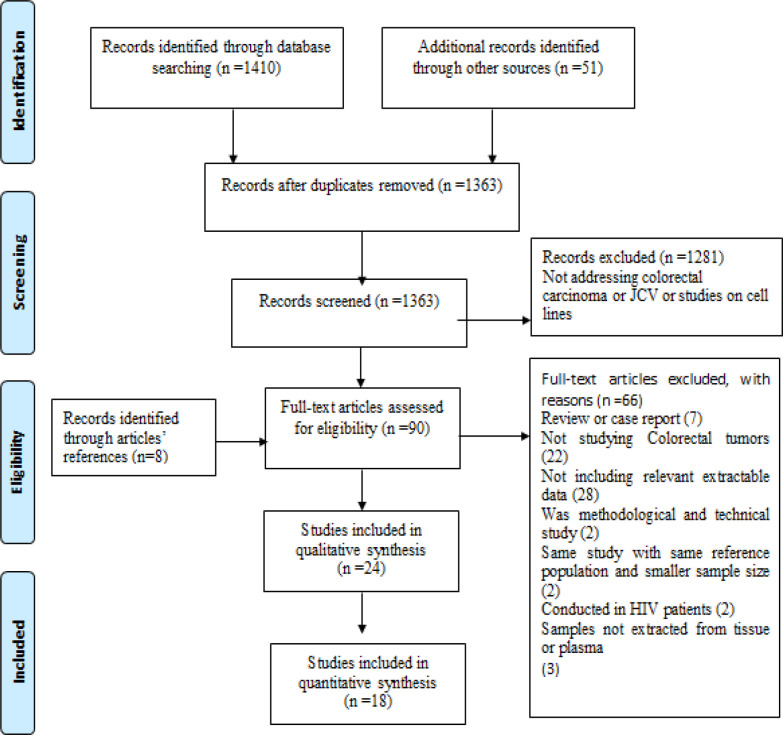
Flow Diagram of the Studies Selected for Systematic Review and Meta-Analysis of JCV and BK viruses in Colorectal Cancer Cases

The initial search resulted in 1,461 articles. After removing 98 duplicates, 1,363 articles underwent title and abstract screening, which yielded the exclusion of 1,281 irrelevant studies. By checking the remained articles (n=82), eight further articles were found through reading the article references. Overall, the eligibility of 90 articles was reviewed by reading of the full text. At this step, 65 articles were excluded for the following reasons: 7 studies were reviewed or case report, 22 studies weren’t conducted on CRC patients, 28 articles didn’t report any relevant extractable data, 2 studies were methodological and technical studies, 1 study was conducted on people living with HIV and 3 studies didn’t used tissue or plasma for sample extraction. A total of 24 articles met the inclusion criteria and were included in qualitative analysis while 18 articles included in quantitative analysis. The flow diagram of study selection process has been shown in Figure 1. 

*Study and participant characteristics*

Majority of included studies were cross-sectional, 28 studies assessed the prevalence of JC virus in CRC (Enam et al., 2002; Hori et al., 2005; Theodoropoulos et al., 2005; Goel et al., 2006; Giuliani et al., 2008; Jung et al., 2008; Lin et al., 2008; Niv et al., 2010a; Niv et al., 2010b; Ramamoorthy et al., 2011; Mou et al., 2012; Matalka et al., 2013; Samaka et al., 2013; Ripple et al., 2014; Ksiaa et al., 2015; Sarvari et al., 2018; Haghi Navand et al., 2019) and five studies assessed the BK virus prevalence (Casini et al., 2005; Giuliani et al., 2008; Tseng et al., 2014; Jarzynski et al., 2017; Sarvari et al., 2018) 

Some studies had used several targets and diagnostic methods (Enam et al., 2002; Hori et al., 2005; Lin et al., 2008; Tsekov et al., 2011; Mou et al., 2012; Matalka et al., 2013; Tseng et al., 2014). One stydy used In Situ Hybridization (ISH) (Samaka et al., 2013), one study used real time PCR(Polymerase Chain Reaction) (Tsekov et al., 2011) and others used PCR, nested-PCR or Immunohistochemistry (IHC), (Enam et al., 2002; Casini et al., 2005; Hori et al., 2005; Theodoropoulos et al., 2005; Goel et al., 2006; Giuliani et al., 2008; Jung et al., 2008; Lin et al., 2008; Niv et al., 2010a; Niv et al., 2010b; Ramamoorthy et al., 2011; Tsekov et al., 2011; Mou et al., 2012; Coelho et al., 2013; Matalka et al., 2013; Ripple et al., 2014; Sinagra et al., 2014; Ksiaa et al., 2015; Jarzynski et al., 2017; Toumi et al., 2017; Sarvari et al., 2018; Haghi Navand et al., 2019). Most of the studies targeted DNA and T antigen (TAg) from the samples in order to diagnose the viruses (Enam et al., 2002; Hori et al., 2005; Goel et al., 2006; Jung et al., 2008; Lin et al., 2008; Niv et al., 2010b; Tsekov et al., 2011; Mou et al., 2012; Vilkin et al., 2012; Matalka et al., 2013; Samaka et al., 2013; Ripple et al., 2014; Tseng et al., 2014; Haghi Navand et al., 2019), however, viral protein 1(VP1), non-coding control region (NCCR) and Agnoprotein were also used as target (Enam et al., 2002; Hori et al., 2005; Tsekov et al., 2011; Tseng et al., 2014). 

*BKV prevalence*

Just five studies had measured the prevalence of BKV in CRC patients (Casini et al., 2005; Giuliani et al., 2008; Tseng et al., 2014; Jarzynski et al., 2017; Sarvari et al., 2018). Because of the low number of studies focusing on BKV by different methods, they were not included in the quantitative analysis. The characteristics of the five studies are shown in table1. The reported prevalence in these studies varies from 0.0% to 100: Casini et al., (2005) that used ISH method found 11 out of 18 cases positive for the virus. By using PCR method, Sarvari et al., (2018) found no virus in 140 of their studies cases, Giuliani et al., (2008) found the virus in 6 out of 66 cases (9%), and Jarzynski et al., (2017) reported 30%(15 of 50 cases). Also Tseng et al., (2014) found BKV in all three cases by using PCR and IHC methods (1 man and 2 women).

*Meta-analysis of JCV prevalence*

**Figure 2:**
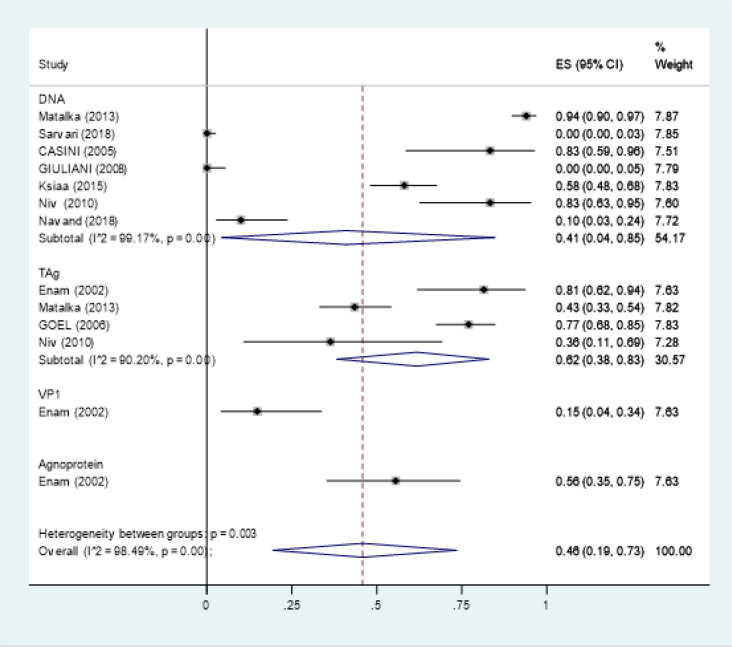
Forest Plot of Meta-Analysis of JCV Prevalence in Colorectal Cancer Ppatients Using PCR as the Method of Detection Categorized by Different Targets (DNA, TAg, VPI, Agnoprotein)

**Figure 3:**
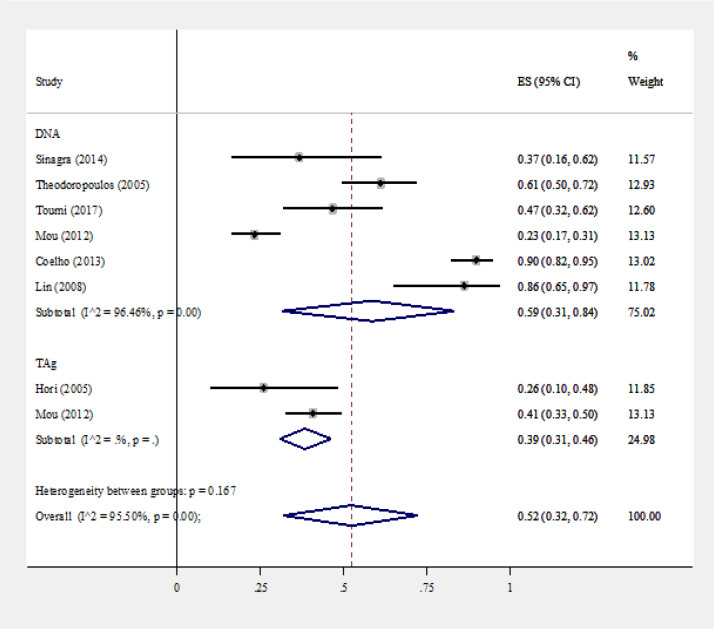
Forest Plot of Meta-Analysis of JCV Prevalence in Colorectal Cancer Patients Using Nested PCR as the Method of Detection Categorized by Different Targets(DNA and TAg)

**Figure 4:**
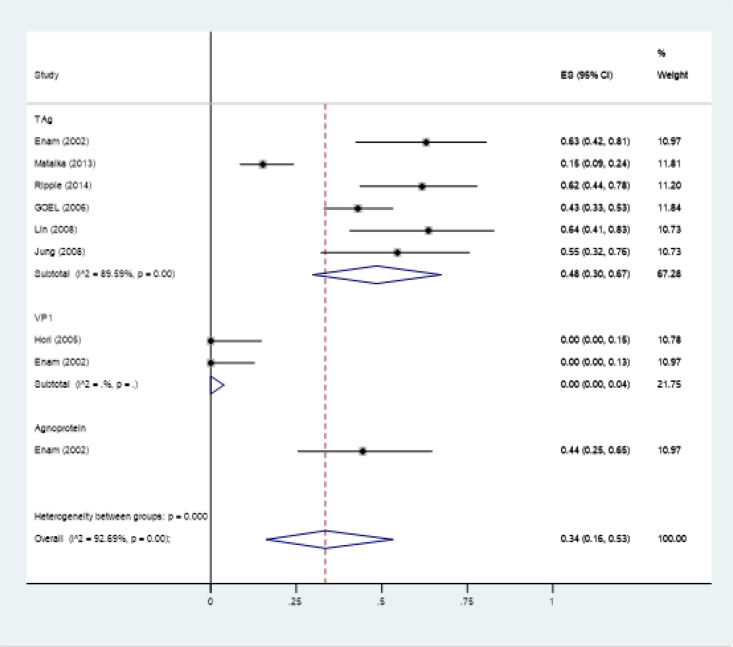
Forest Plot of Meta-Analysis of JCV Prevalence in Colorectal Cancer Patients Using IHC as the Method of Detection Categorized by Different Targets (TAg, VP1, Agnoprotein)

Overall 18 studies reported the JCV prevalence in colorectal cancer that were included in prevalence meta-analysis (Enam et al., 2002; Casini et al., 2005; Hori et al., 2005; Theodoropoulos et al., 2005; Goel et al., 2006; Giuliani et al., 2008; Lin et al., 2008; Niv et al., 2010a; Niv et al., 2010b; Tsekov et al., 2011; Mou et al., 2012; Coelho et al., 2013; Matalka et al., 2013; Sinagra et al., 2014; 2014; Ksiaa et al., 2015; Toumi et al., 2017; Sarvari et al., 2018; Haghi Navand et al., 2019). Nine studies used PCR (Enam et al., 2002; Casini et al., 2005; Goel et al., 2006; Giuliani et al., 2008; Niv et al., 2010b; Matalka et al., 2013; Ksiaa et al., 2015; Sarvari et al., 2018; Haghi Navand et al., 2019), seven studies used nested-PCR (Hori et al., 2005; Theodoropoulos et al., 2005; Lin et al., 2008; Mou et al., 2012; Coelho et al., 2013; Sinagra et al., 2014; Toumi et al., 2017) and seven studies used IHC methods for virus detection). The reported prevalence ranged from 0% to 94% and varied based on the target and diagnostic method (Figures 2-4). 

As the studies were heterogeneous based on method of virus detection, we estimated the pooled prevalence for different detection methods including PCR, nested-PCR and IHC separately. The pooled prevalence (95% confidence interval) was estimated at 46 % (19%- 73%) for PCR, 52% (32%-72%) for nested-PCR and 34 %( 16%- 53%) for IHC (Figures 2-4).

*Prevalence of JCV in subgroups*

There was also a variation in type of target detection in each method. Among studies that used PCR, seven studies used viral DNA (Casini et al., 2005; Giuliani et al., 2008; Niv et al., 2010b; Matalka et al., 2013, Ksiaa et al., 2015; Sarvari et al., 2018; Haghi Navand et al., 2019), 4 studies used TAg(Enam et al., 2002; Goel et al., 2006; Niv et al., 2010b; Matalka et al., 2013) and 1 study used VP1 and Agnoprotein as the target (Enam et al., 2002). In subgroup analysis, the pooled prevalence of JCV in studies used PCR with the target of TAg was 62 % (38%-83%) which was higher than studies used DNA 36% (2%-80%). We didn’t calculate the pooled prevalence for VP1 and Agnoprotein by the PCR method as there was just one study for each of these targets (Enam et al., 2002). The prevalence was reported to be 15 % (4%-34%) for VP1 and 56% (35%-75%) for Agnoprotein (Figure 2). As just one study used real time PCR and NCCR, it was excluded from the metanalysis. In the study 10 and 0 out of 44 cases were positive for the JC virus by using TAg and NCCR, respectively (Tsekov et al., 2011).

From 8 studies used nested-PCR for virus detection, 6 studies used viral DNA (Theodoropoulos et al., 2005; Lin et al., 2008; Mou et al., 2012; Coelho et al., 2013; Sinagra et al., 2014; Toumi et al., 2017), 2 studies used TAg (Hori et al., 2005; Mou et al., 2012) as the target. The pooled prevalence of JCV for DNA target was estimated at 59% (31%- 84%) which was higher than estimated prevalence based on TAg 39% (31%- 46%)) (Figure 3).

From studies used IHC, the TAg, VP1 and Agnoprotein were used as targets in 7, 3 and 1 studies respectively (Enam et al., 2002; Hori et al., 2005; Goel et al., 2006; Jung et al., 2008; Lin et al., 2008; Matalka et al., 2013; Ripple et al., 2014). The pooled prevalence in studies used TAg was 45% (26%- 64%) while 0.0% in studies that used VP1 as the target. We didn’t calculate the pooled prevalence for Agnoprotein as there was just one study for this method. In this study the prevalence was reported to be 44 % (25%-65%) (Figure 4).

*Association of JCV and colorectal cancer*

In 13 studies, the prevalence of JCV in CRC was compared with a control group. There were four types of control groups in order to compare the prevalence of JCV in CRC with them (Casini et al., 2005; Hori et al., 2005; Theodoropoulos et al., 2005; Goel et al., 2006; Jung et al., 2008; Niv et al., 2010b; Tsekov et al., 2011; Mou et al., 2012; Coelho et al., 2013; Matalka et al., 2013; Samaka et al., 2013; Ksiaa et al., 2015; Haghi Navand et al., 2019). The control groups used were healthy people, normal mucous tissue of the same person, patients with colorectal adenoma and patients with non-CRC (HCV, polyp and gastrointestinal disorders other than CRC).

**Figure 5:**
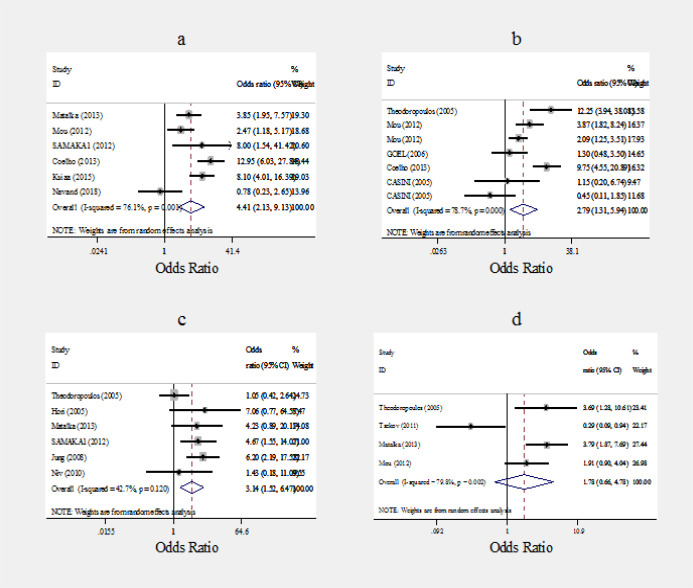
Forest Plot of Associaion between JCV and Colorectal Cancer Presented as Pooled Odds Ratio and 95% Confidence Intevals According to Types of Control Group Used. a, healthy people controls; b, the adjacent mucosa controls; c, adenomatous cases controls; d: Non-colorectal cancer controls

In Figure 5, the pooled odds ratios and 95% confidence intervals comparing JCV in patients with CRC and various types of control groups is shown. The below results are presented as pooled odds ratio (95% confidence intervals). The likelihood of being infected with JCV in CRC cases was significantly higher than all control groups except non-CRC individuals. Compared to healthy controls, CRC patients had 4.41 (2.13 - 9.13) times higher chance of being infected with JCV. Furthermore, the likelihood of being infected with JCV in CRC patients was 3.1 (1.5-6.5) times more than patients diagnosed with colorectal adenoma. Comparing CRC tissue and normal adjacent tissue in the same person, the chance of being infected with JCV in CRC tissue was 2.79 (1.3-5.9) times more than normal adjacent tissue. However, when non-CRC patients used as control group the difference was not significant.

Meta-regression test was used to assess the effect of as type of diagnosis test, location of sampling and sample type on heterogeneity.

In the naive model, without any variable, I2 was 98%. Several models with different variables were made, in which I^2^ ranged from 98% to 99%, and the input of different variables did not have any effects on reducing the amount of heterogeneity.

*Assessing the publication bias*

**Figure 6:**
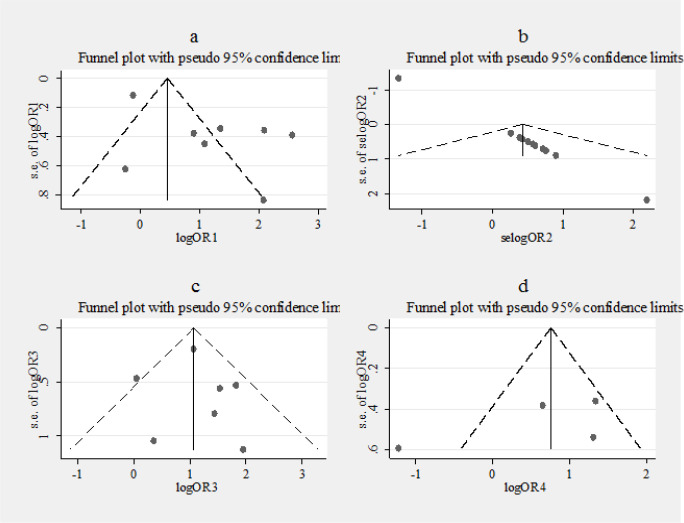
Funnel Plot of Studies Included in the Meta-Analysis of Association between JCV and Colorectal Cancer According to Types of Control Group Used. a, healthy people controls; b, the adjacent mucosa controls; c, adenomatous cases controls; d, Non-colorectal cancer controls

The Egger’s test was non-significant for the pooled odds ratio over the studies that had compared cancerous tissues to adjacent mucosa, adenomatous and NCRC samples while, it was significant for pooled odds ratio over the studies that had compared the cancerous tissues to healthy individuals’ tissues as control group (p value: 0.013). The funnel plot confirmed the Egger’s test results (Figure 6).

**Table 1:** Characteristics of Studies Included in Systematic Review and Meta-Analysis of JCV and BK viruses in Colorectal Cancer Cases

Author/year	Location		Study Design		Number of participants(male/female)		Prevalence											Age of study population		Type of virus/target		Control type		Cancer type		Detection test	
							PCR			Nested-PCR		IHC		Real Time PCR		ISH											
Sinagra et al. 2014(Sinagra et al., 2014)	Italy		Cohort		7-12					DNA:0.37								Median 70 (59-87)		JC/DNA		Non-CRC(HCV)		Colorectal		Nested-PCR	
Tsekov et al. 2011(Tsekov et al., 2011)	Bulgaria		Cross-Sectional		29/15									TAg:0.24 NCCR:0.0				(Mean, range 65; 35-82)		JC/TAg & NCCR		Non-CRC Motivated Strategies for Learning Questionnaire (MSLQ (polyp)		Colorectal		Real time PCR	
Theodoropoulos et al 2005(Theodoropoulos et al., 2005)	Greece		Cross-Sectional		38/42					DNA:0.61								(Mean 62.75), (range, 35–82) (SD± 12.47)		JC/DNA		Adjacent mucosa- adenomatous- Non-CRC		Colon		Nested PCR	
Matalka, 2013(Matalka et al., 2013)	Jordan		Cross-Sectional		44/48		DNA:0.94 TAg:0.43					TAg:0.15						Ages ranged from 20 to 86 years		JC/ TAg &DNA		Adenomatose, Non-CRC		Colorectal		PCR, IHC	
Hori et al.2005(Hori et al., 2005)	Japan		Cross-Sectional		13/10					TAg:0.26		VP1:0.0						Average age of 66.3 years (range40-85)		JC/ TAg& VP1		Adenomatous & Non-CRC		Colorectal		Nested PCR & IHC	
Tae Jung,2008(Jung et al., 2008)	USA		cross -sectional		22(both sex)							TAg:0.55						----		JC/TAg		Adenomatose, Adjacent tissue		Colorectal		PCR& IHC	
Niv. 2010(Niv et al., 2010b)	Israel		cross -sectional		11(both sex)		TAg:0.36											66.9±11.1		JC/TAg		Adenomatose		Colorectal		PCR	
Toumi et al. 2017(Toumi et al., 2017)	Tunisia		Cross-Sectional		24/23					DNA:0.47								(Mean age: 64-years)		JC/DNA		Adjacent mucosa- Non-CRC		Colorectal		Nested PCR	
Tseng et al. 2014(Tseng et al., 2014)	Taiwan		Cross-Sectional		1/2(JC)		DNA:0.0					TAg:0 VP1:0						Renge 65-84		JC/DNA &TAg & VP1		Adjacent mucosa		Colon		PCR &	
Casini,2005(Casini et al., 2005)	Italy		Case-control		18(both sex)		DNA:0.83									DNA:0.50		-----		JC/DNA		Adjacent tissue		Colorectal		PCR & ISH	
Lin,2008(Lin et al., 2008)	Taiwan		Cross -sectional		13-Sep					DNA:0.86		TAg:0.64						47-89		JC/DNA& TAg (large tumor)		Adjacent tissue		Colon cancer		Nested PCR& IHC	
Enam,2002(Enam et al., 2002)	USA		Cross -sectional		17-Oct		TAg:0.81 VP1:0.15 Agnoprotien:0.56					Agnoprotien:0.44 TAg:0.63 VP1:0.0						Range (41-91)		JC/TAg&VP1&Agnoprotein antibody		Adjacent tissue		Colon		PCR& IHC	
Vilkin . 2012(Vilkin et al., 2012)	Israeli		Cross-Sectional		13/17 history& 13/13without history		TAg:0.20											80.9 ± 5.6 histories & 51.7 ± 6.2without history		JC/ TAg		Totally 2 groups(Family history & without a family history)		Colorectal		PCR	
Mou, 2012(Mou et al., 2012)	China		Cross-Sectional		74/63					DNA:0.23 TAg:0.41								Mean age in CRC patients 63 Non-CRC patients 49.6, Healthy donors 62.6		JC /TAg & DNA		Healthy people, Adjacent tissue, Non-CRC		Colorectal		Nested PCR	
Samaka,2012 (Samaka et al., 2013)	Egypt		Cross - sectional		23/34											TAg:0.66		47.8 ± 13.7		JC/ TAg		Healthy people, Adenomatose		Colorectal		ISH	
Samaka,2012 (Samaka et al., 2013)		Egypt		Cross - sectional		23/34									TAg:0.66		47.8 ± 13.7		JC/ TAg		Healthy people, Adenomatose		Colorectal		ISH	
Rasteiro Coelho,2013(Coelho et al., 2013)		Portugal		Case-control		67/33			DNA:0.90								----		JC/DNA		Healthy people Adjacent tissue		Colorectal		Nested PCR	
Ksiaa, 2015(Ksiaa et al., 2015)		North Africa		Cross -sectional		60/45		DNA:058									Mean age 62 years (range 20-95).		JC/DNA		Healthy people		Colorectal		PCR	
Navand1,2018(Haghi Navand et al., 2019)		Iran		Cross -sectional		20/20		DNA:0.10									----		JC/DNA		Healthy people		Colorectal		PCR	
Ripple,2014(Ripple et al., 2014)		USA		Cross-Sectional		26/8					TAg:0.62						35-76		JC/ TAg		--		Colorectal		IHC	
Sarvari,2018(Sarvari et al., 2018)		Iran		Cross - sectional		77/63		DNA:0.0									52 ± 1.64		JC / DNA		--		Colorectal		PCR	
Goel, 2006(Goel et al., 2006)		USA		Cross - sectional		100(both sex)		TAg:0.77			TAg:0.43						----		JC/TAg		--		Colorectal		PCR & IHC	
Giuliani,2008(Giuliani et al., 2008)		Italy		Cross -sectional		66(both sex)		DNA:0.0									----		JC /DNA		----		Colorectal		PCR	
Niv, 2010(Niv et al., 2010a)		Israel		Cross -sectional		24(both sex)		DNA:0.83									71.3±12.6		JC/DNA		--		Colorectal		PCR	
Sarvari,2018(Sarvari et al., 2018)		Iran		Cross - sectional		77/63		DNA:0.0									52 ± 1.64		BK/ DNA		--		Colorectal		PCR	
Giuliani,2008(Giuliani et al., 2008)		Italy		Cross -sectional		66(both sex)		DNA:0.10									----		BK/DNA		----		Colorectal		PCR	
Jarzynski,2017(Jarzynski et al., 2017)		Poland		Cross - sectional		24/26		DNA:0.30									40 years and more		BK/DNA		----		Colorectal		PCR	
Casini,2005(Casini et al., 2005)		Italy		Case-control		18(both sex)									DNA:0.0		-----		BK/DNA		Adjacent tissue		Colorectal		PCR & ISH	
Tseng et al. 2014(Tseng et al., 2014)		Taiwan		Cross-Sectional		2-Jan					VPI:0.0 TAg:1.0						Renge 65-84		BK /DNA &TAg & VP1		Adjacent mucosa		Colon		PCR &IHC	

## Discussion

The results of present systematic review and meta-analysis showed a huge variation regarding the JCV and BKV prevalence in different studies, from no positive case to considerable prevalence by some studies. Furthermore, we showed a positive association between JCV infection and CRC.

The pooled prevalence of JCV was highest when Nested-PCR was used as method of detection and was lowest when IHC was used (52% vs. 29%). These variations may be due to the sensitivity of various techniques used. It is well established that nested-PCR is a high sensitive methods of polyomaviruses detection (Mou et al., 2012a) and as we expected, the polled prevalence of JCV was higher when nested-PCR was used. 

For BKV prevalence, various methods of detection including PCR, IHC and ISH resulted in various prevalence from non-positivity to all-positivity. Furthermore, there are huge variations in estimated prevalence reported by studies in which similar methods were used. For example, Tseng et al., (2014) found all three sample positive by IHC method while none of the three sample were positive when PCR method was used. In contrast, Sarvari et al. found no positive sample using PCR (Sarvari et al., 2018) while Giuliani found 9 percent of sample positive using the same method (Giuliani et al., 2008). As just five studies reported the prevalence of this virus, no valid interpretation could be drawn. 

Behind the heterogeneity between the studies due to applied techniques and targets, other factors may also explain different prevalence obtained by various studies. Environment, geographic area, age and life style are potential factors affecting the prevalence. The primary infection with BKV occurs in early years of life while infection with JVC occurs at older ages: late childhood and adolescence. Seroconversion is observed at highest rates during adolescence and continues at a lower frequency until the age of about 60 (Comar et al., 2012). Environment is another factor affecting the prevalence. Although the transmission mechanism has not clearly explained, the high resistance of the viruses to environmental inactivation and its presence at high concentrations in human sewage and other water sources indicate fecal-oral transmission (Bofill-Mas and Girones, 2001; Bofill-Mas and Girones, 2003; Auer et al., 2018; Levican et al., 2018). JCV could be orally ingested and might infect people through the gut mucosa as it seems to be relatively stable to acidic environment and proteinase exposure present in the gastrointestinal tract (Bofill-Mas and Girones, 2003).Therefore, variation in environmental health in various regions may affect the prevalence. Various studies estimated the prevalence of Polyoma viruses in general population. In Kuwait, 2013, the prevalence of infection with polyomaviruses including JCV and BKV among healthy kidney donors reported to be 42% using semi nested-PCR (Chehadeh et al., 2013). In Pakistan, 2017 the BKV and JCV was detected in 27.1% and 11.6% of healthy individuals respectively using real time PCR (Hussain et al., 2017). Furthermore, Vanchiere et al. in the USA, detected the BKV and JVC in 8% and 9% of stool specimens of healthy individuals using PCR method (Vanchiere et al., 2009). Because of variation in geography, detection methods and back ground characteristics of studied sample, comparing the pooled-prevalence we calculated for CRC patients with general population maybe misleading and therefore we avoid it. To make a valid comparison, high quality studies comparing the polyomaviruses prevalence in CRC patients and healthy individuals from the same demographic back ground and using similar detection method is needed.

We showed a positive association between JVC and CRC. This significant association was observed in 3 types of control groups including healthy people, adjacent normal mucosa and colorectal adenoma. The strength of association was more when healthy people were used as control group than when adjacent normal mucosa and adenomatous tissue considerd. This was expectable because the adjacent and tumour tissues in the same person contain the same genetic message (Wacholder et al., 1992), so the similarity between the cancerous tissue and the adjacent tissue might lead the odds ratio to artificially decrease. Although the carcinogenic effects of JVC has been confirmed in experimental animals, the results in human are controversial. International Agency for Cancer Research Monograph Working Group, in 2012 classiﬁed the JVC as “Group 2” which refers to “possibly carcinogenic to human”(Bouvard et al., 2012). Experimental studies indicate that oncogenic effects of JVC is mainly related to the viral large T antigen which was also one of the most common used primer targets for virus detection in the included studies. The T antigen can inactivate tumor suppressor proteins p53 and pRB (Dalianis and Hirsch, 2013). Furthermore, abnormal DNA methylation and chromosomal instability are other mechanisms that has been attributed to the oncogenic effect of JVC (Chen et al., 2015).

We should state that the associations we found in present meta-analysis should be interpreted with caution. The funnel plot is suggestive of some degrees of publication bias, which means that the calculated odds ratio in the meta-analysis may overestimate the true association between JCV and CRC. In addition, most studies suffer from limitations such as low sample size and inappropriate adjustment of potential confounders. Furthermore, as the temporal sequence of infection with JVC and incidence of CRC wasn’t addressed in any study, the observed association in present study cannot be indicative of causation. JCV infection is usually an asymptomatic infection and commonly occurs in later childhood and adolescence (Chehadeh et al., 2013). Then after, the virus remains latent. Previous studies showed that prevalence of infection with JVC is significantly higher in immunosuppressed people compared to non-immuno-suppressed (Niv et al., 2010a; Niv et al., 2010b) which suggests the reactivation of polyomaviruses following immunosuppressive treatment. So, reverse causation, which refers to the activation of virus after chemotherapy induced immune-suppression in CRC patients is another explanation in this regard. 

In conclusion, JCV and BK infections seems to be common in CRC patients and might be a risk factor for CRC. Large population-based prospective and in vivo studies are needed to determine whether JCV and BK are causative agents of CRC. Improving the life style and environmental health care during early life might decrease the prevalence of the viruses.

## References

[B1] Abend JR, Jiang M, Imperiale MJ (2009). BK virus and human cancer: innocent until proven guilty. Semin Cancer Biol.

[B2] Auer M, Bsteh G, Hegen H (2018). Smoking is not associated with higher prevalence of JC virus in MS patients. Eur J Clin Microbiol Infect Dis.

[B3] Bangham CR, Ratner L (2015). How does HTLV-1 cause adult T-cell leukaemia/lymphoma (ATL)?. Curr Opin Virol.

[B4] Bofill-Mas S, Girones R (2003). Role of the environment in the transmission of JC virus. J Neurovirol.

[B5] Bouvard V, Baan RA, Grosse Y (2012). Carcinogenicity of malaria and of some polyomaviruses. Lancet Oncol.

[B6] Bray F, Ferlay J, Soerjomataram I (2018). Global cancer statistics 2018: GLOBOCAN estimates of incidence and mortality worldwide for 36 cancers in 185 countries. CA Cancer J Clin.

[B7] Casini B, Borgese L, Del Nonno F (2005). Presence and incidence of DNA sequences of human polyomaviruses BKV and JCV in colorectal tumor tissues. Anticancer Res.

[B8] Chan DS, Lau R, Aune D (2011). Red and processed meat and colorectal cancer incidence: meta-analysis of prospective studies. PLoS One.

[B9] Chehadeh W, Kurien SS, Nampoory MR (2013). Molecular characterization of BK and JC viruses circulating among potential kidney donors in Kuwait. Bio Med Res Int.

[B10] Chen H, Chen XZ, Waterboer T (2015). Viral infections and colorectal cancer: a systematic review of epidemiological studies. Int J Cancer.

[B11] Coelho TR, Gaspar R, Figueiredo P (2013). Human JC polyomavirus in normal colorectal mucosa, hyperplastic polyps, sporadic adenomas, and adenocarcinomas in Portugal. J Med Virol.

[B12] Comar M, Zanotta N, Croci E (2012). Association between the JC polyomavirus infection and male infertility. PLoS One.

[B13] Dalianis T, Hirsch HH (2013). Human polyomaviruses in disease and cancer. Virol J.

[B14] Enam S, Del Valle L, Lara C (2002). Association of human polyomavirus JCV with colon cancer: evidence for interaction of viral T-antigen and beta-catenin. Cancer Res.

[B15] Girones S, Bofill-Mas R (2001). Excretion and transmission of JCV in human populations. J Neurovirol.

[B16] Giuliani L, Ronci C, Bonifacio D (2008). Detection of oncogenic DNA viruses in colorectal cancer. Anticancer Res.

[B17] Goel A, Li MS, Nagasaka T (2006). Association of JC virus T-antigen expression with the methylator phenotype in sporadic colorectal cancers. Gastroenterology.

[B18] Haghi Navand A, Teimoori A, Makvandi M (2019). Study on JV virus in patients with colon cancer type adenocarcinoma. Asian Pac J Cancer Prev.

[B19] Höcker M, Hohenberger P (2003). Helicobacter pylori virulence factors—one part of a big picture. Lancet.

[B20] Hori R, Murai Y, Tsuneyama K (2005). Detection of JC virus DNA sequences in colorectal cancers in Japan. Virchows Archiv.

[B21] Hussain I, Tasneem F, Umer M (2017). Specific and quantitative detection of Human polyomaviruses BKPyV and JCPyV in the healthy Pakistani population. Virol J.

[B22] Jarzynski A, Zajac P, Zebrowski R (2017). Occurrence of BK Virus and Human Papilloma Virus in colorectal cancer. Ann Agric Environ Med.

[B23] Jung WT, Li MS, Goel A (2008). JC virus T-antigen expression in sporadic adenomatous polyps of the colon. Cancer.

[B24] Ksiaa F, Allous A, Ziadi S (2015). Assessment and biological significance of JC polyomavirus in colorectal cancer in Tunisia. J Buon.

[B25] Levican J, Acevedo M, León O (2018). Role of BK human polyomavirus in cancer. Infect Agents Cancer.

[B26] Liang PS, Chen TY, Giovannucci E (2009). Cigarette smoking and colorectal cancer incidence and mortality: Systematic review and meta-analysis. Int J Cancer.

[B27] Lin PY, Fung CY, Chang FP (2008). Prevalence and genotype identification of human JC virus in colon cancer in Taiwan. J Med Virol.

[B28] Lynch HT, De la Chapelle A (2003). Hereditary colorectal cancer. N Engl J Med.

[B29] Matalka I, Swedan S, Khabaz MN (2013). JC virus in colorectal cancer: where do we stand?. Future Virol.

[B30] Mou XZ, Chen L, Liu FL (2012). Prevalence of JC virus in Chinese patients with colorectal cancer. PLoS One.

[B31] Murphy G, Pfeiffer R, Camargo MC (2009). Meta-analysis shows that prevalence of Epstein–Barr virus-positive gastric cancer differs based on sex and anatomic location. Gastroenterology.

[B32] Narayanan M, Szymanski J, Slavcheva E (2007). BK virus associated renal cell carcinoma: case presentation with optimized PCR and other diagnostic tests. Am J Transplant.

[B33] Niv Y, Vilkin A, Brenner B (2010a). hMLH1 promoter methylation and JC virus T antigen presence in the tumor tissue of colorectal cancer Israeli patients of different ethnic groups. Eur J Gastroenterol Hepatol.

[B34] Niv Abend JR, Jiang M, Imperiale MJ (2009). BK virus and human cancer: innocent until proven guilty. Semin Cancer Biol.

[B35] Ramamoorthy S, Devaraj B, Miyai K (2011). John Cunningham virus T-antigen expression in anal carcinoma. Cancer.

[B36] Ripple MJ, Struckhoff AP, Trillo-Tinoco J (2014). Activation of c-Myc and cyclin D1 by JCV T-Antigen and beta-Catenin in colon cancer. PLoS One.

[B37] Samaka RM, Abd El-Wahed MM, Aiad HA (2013). Does JC virus have a role in the etiology and prognosis of Egyptian colorectal carcinoma?. Acta Pathol Microbiol Immunol Scandinavica.

[B38] Sarvari J, Mahmoudvand S, Pirbonyeh N (2018). The very low frequency of Epstein-Barr JC and BK viruses DNA in colorectal cancer tissues in Shiraz, Southwest Iran. Polish J Microbiol.

[B39] Shannon-Lowe C, Rickinson AB, Bell AI (2017). Epstein–Barr virus-associated lymphomas. Philosophical Transact Royal Soc B: Biol Sci.

[B40] Sinagra E, Raimondo D, Gallo E (2014). Could JC virus provoke metastasis in colon cancer?. World J Gastroenterol.

[B41] Theodoropoulos G, Panoussopoulos D, Papaconstantinou I (2005). Assessment of JC polyoma virus in colon neoplasms. Dis Colon Rectum.

[B42] Toumi W, Ripalti A, Ricciardiello L (2017). Detection of a new JCV strain of genotype A in a subpopulation of colorectal adenocarcinomas in Tunisia. New Microbiol.

[B43] Tsao SW, Tsang CM, Lo KW (2017). Epstein-Barr virus infection and nasopharyngeal carcinoma. Philos Trans R Soc Lond B Biol Sci.

[B44] Tsekov I, Ferdinandov D, Hristova S (2011). Application Of Real-Time Pcr Technlogies For Analysis Of Jcv As A Human Cancerogen. Biotechnol Biotechnol Equipment.

[B45] Tseng CE, Yeh CM, Fang CY (2014). Detection of human JCPyV and BKPyV in diffuse large B-cell lymphoma of the GI tract. Eur J Clin Microbiol Infect Dis.

[B46] Vanchiere JA, Abudayyeh S, Copeland CM (2009). Polyomavirus shedding in the stool of healthy adults. J Clin Microbiol.

[B47] Vilkin A, Ronen Z, Levi Z (2012). Presence of JC virus DNA in the tumor tissue and normal mucosa of patients with Sporadic Colorectal Cancer (CRC) or with positive family history and Bethesda criteria. Dig Dis Sci.

[B48] Wacholder S, Silverman DT, McLaughlin JK (1992). Selection of controls in case-control studies: II Types of controls. Am J Epidemiol.

